# Who is maintaining weight in a middle-aged population in Sweden? A longitudinal analysis over 10 years

**DOI:** 10.1186/1471-2458-7-108

**Published:** 2007-06-12

**Authors:** Anne N Nafziger, Kristina Lindvall, Margareta Norberg, Hans Stenlund, Stig Wall, Paul L Jenkins, Thomas A Pearson, Lars Weinehall

**Affiliations:** 1 Epidemiology and Public Health Sciences, Department of Public Health and Clinical Medicine, Umeå University, Umeå SE-90185, Sweden; 2 Ordway Research Institute Drug Development Center, Ordway Research Institute, 1365 Washington Avenue, Suite 201, Albany, NY 12206-1066, USA; 3 The Research Institute, Bassett Healthcare, One Atwell Road, Cooperstown, NY 13326-1394, USA; 4 Department of Community & Preventive Medicine, 601 Elmwood Avenue, Box 644, University of Rochester Medical Center, Rochester, NY 14642, USA; 5 National Institute of Public Health – Sweden, Stockholm SE-103 52, Sweden

## Abstract

**Background:**

Obesity has primarily been addressed with interventions to promote weight loss and these have been largely unsuccessful. Primary prevention of obesity through support of weight maintenance may be a preferable strategy although to date this has not been the main focus of public health interventions. The aim of this study is to characterize who is not gaining weight during a 10 year period in Sweden.

**Methods:**

Cross-sectional and longitudinal studies were conducted in adults aged 30, 40, 50 and 60 years during the Västerbotten Intervention Programme in Sweden. Height, weight, demographics and selected cardiovascular risk factors were collected on each participant. Prevalences of obesity were calculated for the 40, 50 and 60 year olds from the cross-sectional studies between 1990 and 2004. In the longitudinal study, 10-year non-gain (lost weight or maintained body weight within 3% of baseline weight) or weight gain (≥ 3%) was calculated for individuals aged 30, 40, or 50 years at baseline. A multivariate logistic regression model was built to predict weight non-gain.

**Results:**

There were 82,927 adults included in the cross-sectional studies which had an average annual participation rate of 63%. Prevalence of obesity [body mass index (BMI) in kg/m^2 ^≥ 30] increased from 9.4% in 1990 to 17.5% in 2004, and 60 year olds had the highest prevalence of obesity. 14,867 adults with a BMI of 18.5–29.9 at baseline participated in the longitudinal surveys which had a participation rate of 74%. 5242 adults (35.3%) were categorized as non-gainers. Older age, being female, classified as overweight by baseline BMI, later survey year, baseline diagnosis of diabetes, and lack of snuff use increased the chances of not gaining weight.

**Conclusion:**

Educational efforts should be broadened to include those adults who are usually considered to be at low risk for weight gain – younger individuals, those of normal body weight, and those without health conditions (e.g. diabetes type 2) and cardiovascular risk factors – as these are the individuals who are least likely to maintain their body weight over a 10 year period. The importance of focusing obesity prevention efforts on such individuals has not been widely recognized.

## Background

Obesity is associated with the development of multiple chronic health conditions and a variety of psychological disorders [1]. In 2002, the Swedish Council on Technology Assessment in Health Care (SBU; Statens beredning för medicinsk utvärdering) estimated that the direct costs for obesity related diseases in Sweden were approximately 2% (approximately 3 billion SEK/year) of the total cost for medical care [2]. Not only is obesity a problem, but increments of increased weight above a normal body weight are also associated with increased risk of numerous health problems[3,4]. Because of these facts, a great deal of effort has been focused on interventions to address the obesity epidemic.

The World Health Organization has recommended prevention of weight gain and promotion of weight maintenance as the first two basic steps in the effective control of obesity [5], but most efforts address secondary and tertiary prevention. While many investigators and public health advocacy groups have tried to develop strategies to assist in weight reduction, fewer have considered how to provide support for weight maintenance [6]. Because public health and health care system efforts in secondary prevention (promotion of weight loss) have been largely unsuccessful, a closer look should be taken at factors related to maintenance of healthy weight as a way to prevent weight gain and obesity.

The prevalence of obesity is increasing rapidly in Sweden [7]. From a primary prevention perspective, shifting the focus from lowering a high risk weight to maintaining low risk weight may be a more effective response to the obesity epidemic. This is not a new concept, but rather a neglected one. Burke et al. have suggested that diabetes prevention (via prevention of weight gain) should target normal and overweight individuals as they make up the majority of incident diabetes cases [8]. The idea that the most benefit can be gained by modest changes among the majority has been applied in the setting of other chronic conditions such as physical inactivity[9,10] and prehypertension[11].

The observation was recently made within a longitudinal study of a Swedish population, that individuals with diabetes type 2 were the most likely to maintain a normal body mass index (BMI) over a ten-year interval [12]. Emmelin et al. found that some survey participants believed they received less attention and less health advice because of a lack of identifiable risk factors [13]. This suggests that the current approach of identifying individuals with a greater risk factor burden and placing the focus primarily on them may be missing those who might get the most benefit from weight maintenance counseling – those who are not yet obese and those without identified cardiovascular risk factors. This led us to explore the characteristics of those who maintain their body weight over time.

The aim of this study was to examine a free-living cohort of middle-aged adults in order to identify and characterize the factors that differentiate individuals who will maintain (or lose) body weight over a 10 year period, from those who will gain, in order to identify those who would benefit from being the target of obesity primary prevention strategies.

## Methods

During the 1970s, Västerbotten County had Sweden's highest cardiovascular disease mortality in ages below 75. These epidemiological data gave rise to a long-term community intervention program, the Västerbotten Intervention Programme (VIP), to reduce major risk factors for cardiovascular disease and diabetes. VIP used both individual- and population-oriented approaches to risk factor reduction [14,15]. Between 1985 and 1991, regional health centers gradually joined the study. By 1990, a uniform protocol for data collection was being used in all participating health centers.

Initially, all citizens were invited to an educational health screening and counseling the year they become 30, 40, 50 and 60 years of age, thus creating annual, consecutive cross-sectional surveys. Because of funding limitations, the County Council discontinued surveys of 30 year olds after 1995, and therefore only 8874 30-year olds contributed data for the cross-sectional analyses from 1990–1995. For the longitudinal surveys, baseline ages were 30, 40 and 50 during 1990–1994 and 10 year follow-up occurred during 2000–2004. Each participant gave written informed consent prior to participation. District nurses conducted the health surveys that included standardized measurements. Weight was measured in light indoor clothing and recorded to the nearest 0.5 kg. Height was measured without shoes and recorded to the nearest centimeter [16]. Blood pressure measurements, fasting blood work and an oral glucose tolerance test were obtained according to standardized procedures [16,17]. Participants completed a questionnaire that included questions on age, education, civil status, use of tobacco products, physical activity, use of certain medications for certain diseases, hypertension, presence of known heart disease and diabetes, and family history of cardiovascular disease and diabetes.

The VIP interventions were designed with both individual interventions and community components [18]. Adults were targeted for screening (via the health survey) and this was accompanied by counseling when cardiovascular risk factors were identified. Because the VIP surveys were conducted in the primary health clinic, the participant's doctor was aware of identified risk factors. At the same time, public health messages about healthy diet and alcohol consumption, smoking, the benefits of physical activity, and healthy psychosocial conditions were being conveyed to the local community. There was a particular focus on reducing dietary saturated fat, as elevated cholesterol was the most prevalent cardiovascular risk factor in the population. The community components were low-budget and designed primarily to use existing community resources. The goal was reduction of CVD risk factors and ultimately CVD [19], and neither obesity prevention messages nor obesity treatments were included.

**Figure 1:**
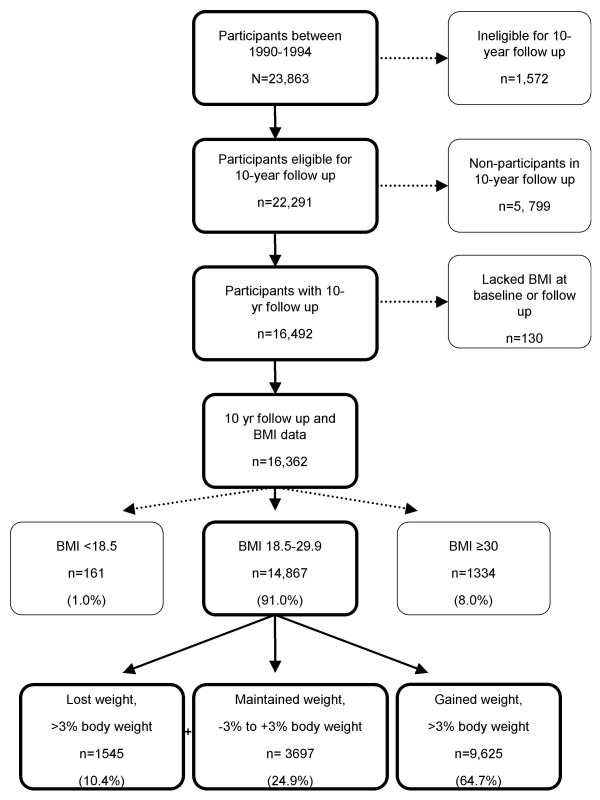
Västerbotten Intervention Programme longitudinal study participants according to body mass index and weight change category.

Participants were categorized according to baseline body mass index (BMI; kg/m^2^). The longitudinal analysis was restricted to those with a baseline BMI of 18.5 to 29.9; those who were underweight (BMI <18.5) or obese (BMI ≥ 30) were excluded (see Figure 1). The remaining participants were then categorized according to weight loss of >3.0%, weight maintenance +/- 3.0%, or weight gain of >3.0% of baseline weight [20]. They were further categorized as non-gainers (weight loss or weight maintenance) or gainers (weight gain).

Impaired glucose tolerance was defined as a fasting capillary plasma glucose of <7.0 mmol/L and a 2-hour capillary plasma glucose after a 75 g glucose load (oral glucose tolerance test) of ≥ 8.9 to <12.2 mmol/L and without a diagnosis of diabetes type 2 [21]. Diabetes mellitus type 2 was defined as self-report, fasting capillary plasma glucose of ≥7.0 mmol/L, or 2-hour capillary plasma glucose (oral glucose tolerance test) of ≥12.2 mmol/L [21]. Hypertension was defined as a mean blood pressure of ≥140/90 mmHg or self-reported use of a medication for hypertension. Hypercholesterolemia was defined as a fasting total cholesterol of ≥7.5 mmol/L. Participants were classified as smokers (yes/no) and snuff users (Swedish moist snuff (snus); yes/no) based on self-report. Family history of myocardial infarction or stroke before the age of 60 years, or family history of diabetes was reported in first degree relatives as "yes/no". A six-level variable was formed to allow comparison by age-sex group (e.g. 30-year old men, 40-year old men, 50-year old men, 30-year old women, etc.) and 30-year old men were used as the reference group.

Prevalences for obesity were calculated from the cross-sectional data and incidences were calculated from the longitudinal data. Chi-square tests were used to compare differences in binomial proportions. Two sample independent t-tests were used to compare continuous variables between groups.

Univariate logistic regression analyses identified significant baseline predictors of weight non-gain. A multivariate logistic regression model was built by adding predictors significant in the univariate analyses, one variable at a time. The model evaluated predictors of weight non-gain over 10 years; gainers vs. non-gainers was the dependent variable. All two-way and three-way interactions were tested. Goodness of fit for the final model was evaluated with Hosmer-Lemeshow test.

The Västerbotten County Council was responsible for maintaining a single database of all the collected data. The Research Ethics Committee at Umeå University approved the study, the National Computer Data Inspection Board approved the data handling procedures, and the study was carried out in compliance with the Helsinki Declaration.

## Results

### Cross-sectional surveys

**Figure 2:**
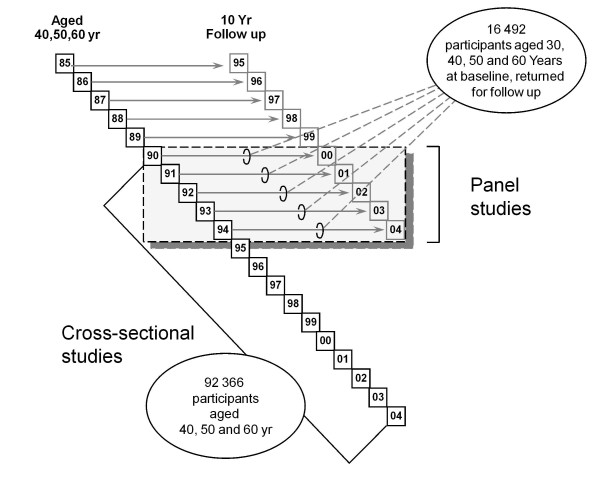
Västerbotten Intervention Programme study design showing survey years, age and number of participants, and cross-sectional and longitudinal study populations.

Between 1985 and 1989, pilot surveys were conducted. Those participants are excluded from this analyses as they were not drawn from the entirety of Västerbotten County. Between 1990 and 2004, 92,366 people participated in the surveys and they are the basis of the cross-sectional survey results presented. See Figure 2. The annual survey response rates averaged 63%. In total, there were 91,801 adults aged 30 to 60 years, including 4361, 13,600, 14,519, and 11,556 men aged 30, 40, 50 and 60 yr, respectively, and 4513, 14,706, 15,617, and 12,929 women aged 30, 40, 50 and 60 yr, respectively. There were a smaller number of 30 year olds because they were only surveyed during 1990–1995, as noted above.

**Figure 3:**
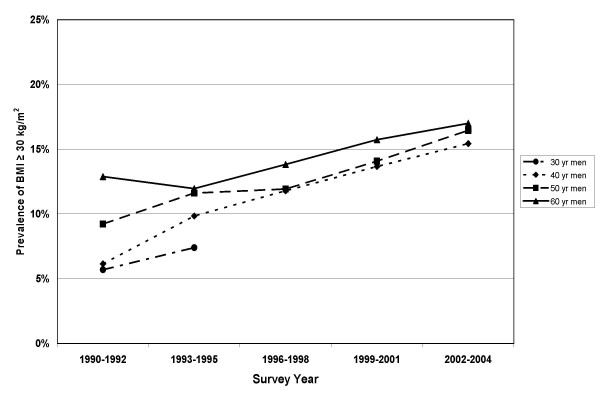
Prevalence of obesity among men participating in the Västerbotten Intervention Programme cross-sectional studies between 1990 and 2004. 30 year olds were not surveyed after 1995.

**Figure 4:**
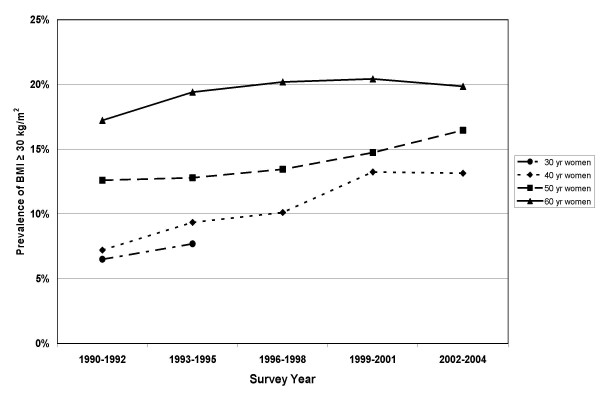
Prevalence of obesity among women participating in the Västerbotten Intervention Programme cross-sectional studies between 1990 and 2004. 30 year olds were not surveyed after 1995.

Prevalence of obesity (BMI ≥ 30 kg/m^2^) progressively increased from 9.4% in 1990 to 17.5% in 2004. Obesity prevalences by sex and 10 year age group are shown in Figure 3 (men) and Figure 4 (women). The 60 year olds had the highest obesity prevalences and the 30 year olds had the lowest. The differences between age groups were greater for the women than for the men. Prevalences of obesity increased for all groups with advancing survey years, except among 60 year old women where the prevalences were stable after 1993 (see Figure 4).

### Longitudinal surveys

Between 1990 and 1994, 23,863 individuals participated in the surveys that formed the basis of the 10-year longitudinal study. Of those, 22,291 were eligible for the longitudinal study and 16,492 participated in the 10-year follow-up survey. See Figure 2. The ineligible included 1062 participants who moved out of the county, 503 individuals who died, and 7 who could not be located because of assignment of an anonymous civil number. The overall follow-up rate was 68.1%; the response rate among the eligible was 74%. Included in this analysis were 14,867 adults [7056 (47.5%) men and 7811 (52.4%) women] who were aged 30, 40 or 50 years at baseline, and had a measured BMI of 18.5–29.9 at the baseline survey. See Figure 1. Participants who lacked a BMI were also excluded. Participants with an initial BMI <18.5 or ≥30 kg/m^2 ^were excluded from this analysis. A comparison of baseline factors for participants versus non-participants is shown in Table 1. The non-participants were more likely to be younger, men, have higher education, have higher serum cholesterol levels, physically inactive, diabetics, and use nicotine.

**Table 1:** Comparison of baseline characteristics for participants and non-participants in the 10-year longitudinal study within the Västerbotten Intervention Programme surveys, 1990–1994.^1^

Baseline characteristic		Participants (%)	Non-participants (%)
Age (years)	30	23.4%	29.9%^§^
	40	39.7%	39.3%
	50	36.9%	30.8%
Sex	Male	46.6%	50.4%^§^
	Female	53.4%	49.6%
Education	Low	22.8%	21.8%^†^
	Medium	53.9%	52.6%
	High	23.4%	25.6%
Body mass index, kg/m^2^		24.8 (3.8)	25.2 (4.1)
Serum cholesterol, mean (SD) mmol/L		5.49 (1.20)	5.52 (1.23)*
Blood pressure, mean (SD) mmHg	Systolic	123 (15)	124 (15)
	Diastolic	77 (10)	77 (11)
Glucose metabolism	Normal	93.2%	92.6%^†^
	Glucose intolerance	4.0%	3.6%
	Diabetes	2.8%	3.7%
Smoker		24.8%	30.4%^§^
Snuff use		25.6%	28.7%^§^
Physically inactive		41.5%	42.9%^†^

^1^This table includes only those eligible for the longitudinal study. *differed by p < 0.05 between participants and non-participants; ^†^differed by <0.01 between participants and non-participants; ^§^differed by <0.001 between participants and non-participants

**Figure 5:**
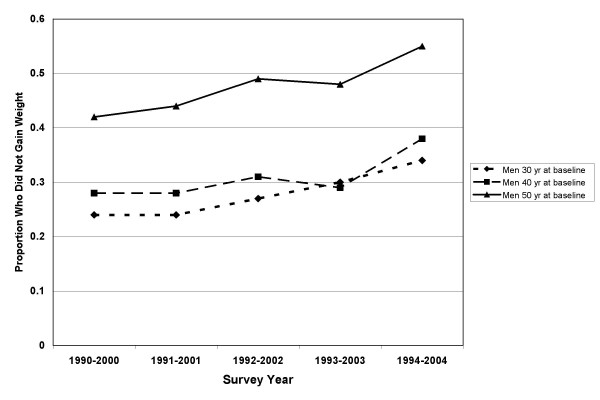
Proportion of men maintaining weight in the Västerbotten Intervention Programme longitudinal studies between 1990 and 2004 by age group.

**Figure 6:**
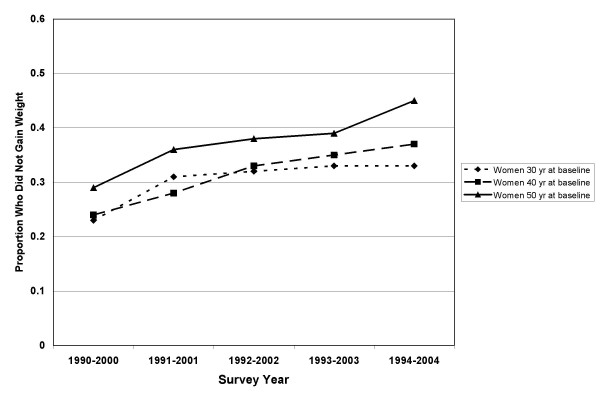
Proportion of women maintaining weight in the Västerbotten Intervention Programme longitudinal studies between 1990 and 2004 by age group.

In the longitudinal study, 5242 adults were categorized as non-gainers (weight gain of <3%) and 9625 were categorized as gainers (weight change of ≥3%). The incidence of overweight was 316/1000. This included those of normal BMI (18.5–24.9 kg/m^2^) who became overweight (BMI 25–29.9 kg/m^2^). The incidence of obesity was 107/1000. This included those of normal or overweight BMI (18.5–29.9) who became obese (BMI ≥ 30 kg/m^2^). BMI, weight, and baseline characteristics by sex are shown for non-gainers and gainers in Table 2. Ten-year percent change in weight ranged from minus 12.0% (1^st ^percentile) to plus 29.5% (99^th ^percentile). The mean (± SD) percent changes in weight were 5.5 ± 6.9 % and 6.6 ± 9.1 % for men and women, respectively. A cohort effect was seen for weight maintenance with greater proportions of people maintaining or losing weight in the later cohorts (Figures 5 and 6).

**Table 2:** Baseline and 10-year follow up characteristics of men and women in the Västerbotten Intervention Programme longitudinal study, 1990–2004, by weight gain or non-gain status.^1^

**Baseline characteristic**		**Men**		**Women**	
		
		**Non-gain**	**Gain**	**Non-gain**	**Gain**
Number (%)		2569 (36.4)	4487 (63.6)	2673 (34.2)	5138 (65.8)
BMI (kg/m^2^)	Baseline	25.1 ± 2.4	24.5 ± 2.5	23.9 ± 2.6	23.5 ± 2.6
	10-year follow-up	24.9 ± 2.7	26.8 ± 3.1	23.4 ± 2.7	26.2 ± 3.6
Weight (kg)	Baseline	79.7 ± 9.1	78.3 ± 9.3	65.2 ± 8.2	64.0 ± 8.0
	10-year follow-up	78.7 ± 9.1	85.6 ± 10.8	63.6 ± 8.1	71.3 ± 10.1
Civil status (%)	Single	13.2	13.2	6.1	6.9
	Married	81.9	80.9	85.9	84.1
	Widowed	0.4	0.4	1.3	1.2
	Divorced or separated	3.4	4.2	5.3	6.6
	Remarried	1.0	1.3	1.4	1.1
Education (%)	Low	28.0	21.9	25.2	22.3
	Medium	50.6	57.2	46.9	49.2
	High	22.3	18.7	26.1	27.0
Serum cholesterol, mmol/L		5.3 ± 4.0	5.5 ± 1.2	5.5 ± 1.1	5.3 ± 1.1
Heart disease (%)		4.3	4.0	2.5	2.0
Use of medicine for heart disease (%)		3.3	3.2	2.5	2.0
Family history of CV disease (%)		17.0	16.6	17.5	18.3
Hypertension (%)		28.5	21.2	19.8	17.2
Snuff use (%)		21.8	26.3	2.4	3.5
Glucose metabolism (%)	Normal	95.5	97.5	93.7	95.2
	Glucose intolerance^2^	3.1	1.9	5.0	1.4
	Diabetes type 2^3^	1.4	0.7	1.3	0.7
Family history of diabetes (%)		15.0	13.2	16.7	16.3

Data are presented as mean (± SD) unless otherwise noted. ^1^Non-gain was defined as weight loss or maintenance of body weight within 3% of baseline weight. Gain was defined as an increase in weight of ≥3%.

In univariate analyses, older age, being a woman, low educational attainment, higher baseline BMI, glucose intolerance or diabetes, hypertension, higher serum cholesterol, lack of current snuff use, and later survey year predicted non-gain. See Table 3.

**Table 3:** Odds ratios for univariate analyses of baseline characteristics and 10-year weight non-gain.^1^

Baseline Characteristic		Odds ratio^2^	(95% CI)
Men	30 yr	1.0	
	40 yr	1.14	(1.00–1.30)
	50 yr	2.36	(2.08–2.68)
Women^3^	30 yr	1.17	(1.02–1.35)
	40 yr	1.18	(1.04–1.34)
	50 yr	1.59	(1.40–1.80)
Civil status	Single	1.0	
	Married	1.04	(0.93–1.16)
	Widowed	1.11	(0.77–1.60)
	Divorced or separated	0.82	(0.68–0.98)
	Remarried	1.00	(.073–1.38)
Education	High	1.0	
	Medium	0.93	(0.86–1.01)
	Low	1.22	(1.11–1.34)
Baseline BMI	18.5–24.9	1.0	
	25.0–29.9	1.27	(1.21–1.34)
Survey year	1990	1.0	
	1991	1.23	(1.08–1.39)
	1992	1.39	(1.23–1.57)
	1993	1.44	(1.27–1.62)
	1994	1.71	(1.52–1.93)
Serum cholesterol, mmol/L		1.14	(1.10–1.17)
Heart disease	No	1.0	
	Yes	1.15	(0.96–1.38)
Use of medicine for heart disease	No	1.0	
	Yes	1.12	(0.92–1.37)
Family history of CV disease	No	1.0	
	Yes	0.98	(0.90–1.07)
Hypertension	No	1.0	
	Yes	1.35	(1.24–1.46)
Cigarette smoker	No	1.0	
	Yes	0.99	(0.92–1.05)
Snuff use	No	1.0	
	Yes	0.82	(0.74–0.91)
Glucose metabolism	Normal	1.0	
	Glucose intolerance^4^	1.35	(1.13–1.60)
	Diabetes type 2^5^	2.05	(1.47–2.85)
Family history of diabetes	No	1.0	
	Yes	1.04	(0.94–1.13)

^1^Weight non-gain included individuals who lost weight and those who maintained their body weight within 3% of the baseline weight. ^2^Using a logistic regression model with weight gain of >3% body weight versus no weight gain. ^3^A six-level age-sex variable was created, using 30 year old men as the reference group. ^4^Fasting glucose <7.0 mmol/L and 2-hour capillary plasma glucose after a 75 g glucose load (oral glucose tolerance test) ≥ 8.9 to <12.2 mmol/L and without diagnosis of diabetes type 2 [21]. ^5^Diabetes mellitus defined as self-report, fasting blood glucose of ≥7.0 mmol/L, or 2-hour capillary plasma glucose (oral glucose tolerance test) of ≥12.2 mmol/L [21].

The final multivariate logistic regression model is presented in Table 4. Older age, being female, classified as overweight by baseline BMI, later survey year and baseline diagnosis of diabetes increased the chances of not gaining weight. Those who did not use snuff also were more likely to be non-gainers. The model showed goodness-of-fit and there were no significant interaction terms.

**Table 4:** Odds ratios for the multivariate model of baseline characteristics and 10-year weight non-gain.^1^

Baseline Characteristic		Number	Odds Ratio (95% CI)^2^
Men	30 yr	1726	1.00
	40 yr	835	1.13 (0.99, 1.30)
	50 yr	2495	2.24 (1.96, 2.56)
Women^3^	30 yr	1787	1.14 (0.98, 1.32)
	40 yr	3152	1.17 (1.03, 1.35)
	50 yr	2872	1.50 (1.16, 1.34)
Body mass index (kg/m^2^)	18.5–24.9	9412	1.00
	25–29.9	5455	1.25 (1.16, 1.34)
Year of initial survey	1990	1802	1.00
	1991	2724	1.19 (1.04, 1.36)
	1992	3465	1.38 (1.21, 1.56)
	1993	3648	1.43 (1.26, 1.62)
	1994	3228	1.74 (1.53, 1.98)
Glucose metabolism	Normal	14238	1.00
	Glucose intolerance^4^	452	1.15 (0.94, 1.40)
	Type 2 diabetes^5^	177	1.47 (1.08, 1.99)
Snuff use	No	12888	1.00
	Yes	1977	0.83 (0.74, 0.92)

^1^Weight non-gain included individuals who lost weight and those who maintained their body weight within 3% of the baseline weight. ^2^Using a logistic regression model with weight gain of >3% body weight versus no weight gain. The model showed goodness-of-fit by Hosmer-Lemeshow test. There were no main effect interactions. ^3^A six-level age-sex variable was created, using 30 year old men as the reference group.^4^Fasting glucose <7.0 mmol/L and 2-hour capillary plasma glucose after a 75 g glucose load (oral glucose tolerance test) ≥ 8.9 to <12.2 mmol/L and without diagnosis of diabetes type 2 [21]. ^5^Diabetes mellitus defined as self-report, fasting blood glucose of ≥7.0 mmol/L, or 2-hour capillary plasma glucose (oral glucose tolerance test) of ≥12.2 mmol/L [21].

## Discussion

### Cross sectional surveys

Using cross-sectional data, we can calculate the prevalence of normal BMI in the population (or in contrast, the prevalence of obesity). The cross-sectional data suggest a gradual, steady increase in the prevalence of obesity within this middle-aged population during the time period from 1990–2004. An increase in the mean weight, with a resultant increase in obesity prevalence, was common in each of the sex and age groups studied. Among the women, those 40 and 50 years old had increasing prevalences of obesity but the 60 year old women had a relatively stable prevalence. In the U.S., the prevalence of obesity in women has been noted to be leveling off [22] while continuing to increase in men and children. This is consistent with our finding that women have a higher likelihood of maintaining body weight. A study in 70-year old Swedes also found that prevalences of obesity were higher in later cohorts[23].

### Longitudinal surveys

By employing longitudinal data, we can identify the characteristics of those who are maintaining or losing weight. The longitudinal study reveals a different picture of the obesity epidemic and those who are avoiding weight gain than do the cross-sectional surveys. Overall, only 35% of the middle-aged population who were normal or overweight was able to maintain or lose body weight over ten years, with the remainder becoming heavier. The 30 yr olds were least likely to maintain or lose weight. There are numerous reasons why 30 year olds might have an increased risk of gaining weight. These include pregnancy [24], changes in lifestyle related to career development (e.g., working overtime) [25], or dissatisfaction with the need to combine work and family life [25]. Our data do not allow us to explore these types of underlying reasons.

Individuals with glucose intolerance identified during their baseline survey, or those with diabetes type 2, were the most likely to avoid weight gain. Increased body weight is a well recognized risk factor for diabetes [8], and the majority of individuals with diabetes type 2 are overweight or obese. The observation that individuals with diabetes were less likely to gain weight over the ten year interval may be attributable to increased intervention efforts (i.e. secondary prevention) on the part of the primary care system or to poorly controlled diabetes. Similar results have been reported by others [26]. The mean fasting glucose among diabetics was only 7.2 mmol/L, making poorly controlled diabetes an unlikely explanation for an increased probability of non-gain. Since the best way to prevent diabetes is likely by preventing weight gain among normal weight and overweight people [8], from a public health perspective it is preferable to have effective primary prevention strategies and therefore have reduced need for secondary prevention efforts.

The classification of who is maintaining weight in this study may be generous. We chose to use the recommendations of Steven et al[20] because of the importance of publishing data that can be compared to the work of others. Definitions of weight maintenance and weight change are often not standardized between studies. Using cut points based on gain of < 3% body weight should allow for differences in body size, measurement error, clothing weight and fluid balance [20], and change in height (e.g. with aging).

A limitation of the current study is that participation rates were not optimal. In a separate analysis from the Swedish Census, the differences were minimal with regard to education, income and employment types between participants and non-participants[27]. However, in the longitudinal study the participants were more likely to be of older age, women, lower education, lower baseline BMI and less likely to have cardiovascular risk factors. Overall, the differences between participants and non-participants should have resulted in more conservative odds ratios in our final model.

### Community intervention effects versus secular trends

We noted a cohort effect, with a higher proportion of each age group maintaining weight as time progressed (Figures 5 and 6). This could mean that a slowing of the obesity epidemic is occurring but is not yet evident in the cross-sectional prevalence. The higher numbers of weight maintainers may also be the consequence of local community interventions to reduce cardiovascular disease risk.

The population studied in VIP has been participating in a program to reduce cardiovascular risk since the early 1990s. Programs intended to reduce cardiovascular risk could reduce obesity risk, although this has never been shown. Other community intervention programs for cardiovascular disease risk have been successful in altering cardiovascular risk but have failed to prevent weight gain [28-30]. The VIP interventions to encourage low fat diets, increase physical activity and control of other cardiovascular risk factors may provide an explanation for our findings. However, it should be noted that the goal of decreasing dietary saturated fat was intended to lower serum cholesterol, not reduce caloric intake. In addition, obesity as an important Swedish health problem, has only recently received wide recognition [31]. VIP was designed and implemented at the local, primarily rural, level and has been sustained for more than 20 years. This duration is longer than others with such a large intervention group. A longer, sustained intervention may be more successful although effects on weight gain have not been specifically evaluated.

One explanation for our findings may be that those with a chronic disease or identified risk factor are more attentive to weight maintenance and weight loss, while those without risk factors are not placing a priority on maintaining or losing weight. An alternative explanation may be that there are secular changes taking place that are slowing weight gain but that are not necessarily the direct result of interventions. A third possibility is that public health programs and medical care focus educational efforts on those with risk factors or chronic diseases rather than those who are perceived to be healthy (the young and lean) and therefore thought to be at low risk for future health problems.

### Unexpected weight maintainers

We expected that individuals with normal BMI at baseline would be the most likely to maintain their body weight. Instead, we found that it is the people with normal body weight who are most likely to gain. Being younger, leaner and free of health problems increases the risk for weight gain by up to 150%. The finding that older adults are more likely to maintain weight is not unique to our population. An epidemiologic study from Norway also found that weight maintenance is more likely among those who are of older age [32]. Being overweight was associated with lower risk of weight gain, possibly because these individuals have been identified as at risk and are the recipients of secondary prevention from public health interventions or health care providers. The association between glucose intolerance identified at baseline and lack of weight gain supports this interpretation. Another possibility is that those who are overweight are paying more attention to their weight and/or working to avoid weight gain.

## Conclusion

Efforts to assist people in losing weight have been largely unsuccessful [33,34]. Because increases in BMI above the normal range are associated with increases in the risk of many health conditions, poorer quality of life and mortality [35], the time to intervene is before weight gain occurs. Researchers and public health officials have been actively working to identify interventions that can be globally implemented for population-wide changes to dietary behaviors and physical activity [36]. In the U.S., there have been programs supported by the government, academic research community, and public health organizations but none have been successful in curtailing obesity [37].

The suggestion has been made that the public should learn about the body weight that is optimal for health, although it is unclear what type of educational programs should be implemented. By its longitudinal design, VIP gives further opportunities to provide data on weight maintenance, and thereby adds on to our present base of knowledge of what kind of preventive initiatives are likely to contribute and support people to maintain an optimal body weight. A developed perspective on maintaining weight in primary prevention does not replace or decrease secondary prevention efforts, but should rather be seen as a complement.

Educational efforts should be broadened to include those adults who are usually considered to be at low risk for weight gain – younger individuals of normal body weight, and those who are without health conditions (e.g. diabetes type 2) or cardiovascular risk factors – as these are the individuals who are least likely to maintain their body weight over a 10 year period. This does not negate the importance of continuing effective secondary prevention strategies. The importance of preventing weight gain earlier in life rather than later has been emphasized by others [5,8], but the importance of focusing obesity prevention efforts on those without risk factors has not been widely recognized.

## List of abbreviations used

BMI, body mass index

VIP, Västerbotten Intervention Programme

## Declaration of competing interests

The author(s) declare that they have no competing interests.

## Authors' contributions

ANN conceived the study, carried out the statistical analyses and drafted the manuscript. KL and MN provided critical revisions. HS and PLJ provided guidance on the statistical analyses. SW and LW conceived the study and provided critical review. TAP provided conceptual guidance and critical review.

## Pre-publication history

The pre-publication history for this paper can be accessed here:


